# The Influence of Induced Head Acceleration on Lower-Extremity Biomechanics during a Cutting Task

**DOI:** 10.3390/s24155032

**Published:** 2024-08-03

**Authors:** Warren O. Forbes, Janet S. Dufek

**Affiliations:** 1 Kinesiology Department, California State University San Bernardino, San Bernardino, CA 92407, USA; 2 Department of Kinesiology and Nutrition Sciences, University of Nevada Las Vegas, Las Vegas, NV 89154, USA; janet.dufek@unlv.edu

**Keywords:** concussion, mild traumatic brain injury, landing, kinematics, kinetics

## Abstract

Sports-related concussions are caused by one substantial impact or several smaller-magnitude impacts to the head or body that lead to an acceleration of the head, causing shaking of the brain. Athletes with a history of sports-related concussion demonstrate lower-extremity biomechanics during landing tasks that are conducive to elevated injury risk. However, the effect of head acceleration on lower-extremity biomechanics during landing tasks is unknown. Twenty participants were evenly separated into a vertical hopping group and a lateral hopping group. Participants performed several land-and-cut maneuvers before and after a hopping intervention. Vertical head acceleration (g) was measured via an accelerometer during the hopping interventions. Comparisons in head acceleration during the hopping tasks were made between groups. Additionally, kinematic and kinetic variables were compared pre- and post-intervention within groups as well as post-intervention between groups. The vertical hopping group demonstrated greater vertical head acceleration compared to the lateral hopping group (*p* = 0.04). Additionally, the vertical hopping group demonstrated greater knee abduction angles during landing post-intervention compared to the lateral hopping group (*p* < 0.000). Inducing head acceleration via continuous hopping had an influence on lower-extremity biomechanics during a landing task.

## 1. Introduction

Sports-related concussions (SRCs) play a very debilitative role in athletics and cause a considerable financial impact on the US healthcare system [1]. Current return to play protocols typically involve a set of clinical examinations that increase in cognitive and exercise intensity as one satisfactorily progresses through the protocol [2,3]. Much focus during the return to play process is centered around restoring cognition to pre-SRC levels as well as ensuring no SRC-related symptoms persist during sports-specific activities. Most athletes are able to return to their sport within 1–3 weeks post-injury [2,3]; however, long-term neurophysiological and motor impairments may still be present beyond the return to play timeframe [4,5]. These impairments can lead to an increased likelihood for an athlete who has suffered an SRC to sustain a musculoskeletal injury upon their return to sport. In the recent literature, it has been suggested that college athletes who suffer an SRC are up to 3.7 times more likely to sustain a musculoskeletal injury compared to athletes without a history of a SRC [6,7,8,9,10,11]. Lower-extremity injuries are of particular concern following an SRC, as athletes are as high as 10% more likely to sustain a non-contact lower-extremity injury compared to athletes without an SRC history [6,9,10,11]. While any connection between SRC and lower-extremity injury is not clearly understood, the recent literature suggests that athletes with a history of a SRC perform high-impact landing-related tasks with decreased lower-extremity neuromuscular control compared to healthy control athletes [6,12,13,14,15,16,17]. 

SRCs can be characterized as a form of mild traumatic brain injury. The Centers for Disease Control and Prevention state that a mild traumatic brain injury is caused by a bump, blow, or jolt to the head, or by a hit to the body that causes the head and brain to accelerate back and forth [18]. While the Centers for Disease Control and Prevention definition is applied to the broad umbrella of mild traumatic brain injury, the same mechanisms pertain to SRCs. The mechanisms for SRCs include a combination of linear and angular accelerations of the head, with the only additional characteristic being that the accelerations were applied to the head specifically in a sports setting [19]. An SRC can occur due to one substantial impact causing an acceleration of the head or can be the result of several lower-magnitude impacts resulting in a multitude of head accelerations, termed sub-concussive loads [20]. While the current literature has examined the influence of sub-concussive loads on inducing an SRC, the link between repeated head acceleration and lower-extremity biomechanics during high-impact landing-related tasks has not been examined. Since it is not feasible nor ethical to induce participants with a sub-concussive load, this study measured head acceleration values during a continuous hopping activity. Continuous hopping was used as the experimental perturbation since it is able to cause several consecutive head accelerations. Furthermore, the task is safe, practical, and easy to administer. While the magnitude of head acceleration during continuous hopping is typically not enough to be characterized as a sub-concussive load, if increased head accelerations exhibited during the continuous hopping task can elicit a perturbation to the system that temporarily alters landing mechanics, then this would coincide with current findings in the concussion literature where an SRC leads to decreased neuromuscular control during high-impact landing tasks. It is possible that repeated acceleration of the head and brain can lead to neuromuscular control deficits that can influence landing biomechanics and increase lower-extremity injury risk. If the negative effects of an SRC on landing mechanics are similarly present after a surrogate head-acceleration-inducing task such as continuous hopping, then this can provide a safer alternative for future SRC research measuring neuromuscular control during landing. Therefore, this study serves to assess whether the induced head acceleration via a continuous hopping task leads to a substantial enough perturbation to the system to elicit a temporary neuromuscular response during subsequent landing tasks. This study also served to address a current gap in the literature as to whether there is a relationship between the magnitude of head acceleration and lower-extremity mechanics during landing-related tasks. The overall purpose of this study was to examine the influence of vertical head acceleration values measured during a uniquely directional continuous hopping activity on lower-extremity biomechanics during a land-and-cut task. 

## 2. Materials and Methods

### 2.1. Participants

Due to the novelty and exploratory nature of this study, the target population of interest was expanded beyond the realm of athletes. Twenty healthy individuals (1.7 ± 0.1 m, 77.85 ± 35.0 kg, 22.6 ± 3.4 yrs) volunteered for this study. Inclusion criteria were as follows: between 18–30 years of age, ability to participate in physical activity such as continuous jumping and landing, exercise at least one time per week for a minimum of one hour, not currently pregnant, no concussion history within the past five years, and no current lower-extremity injuries that would limit participation in the study. Participants were counterbalanced to one of two groups: (1) continuous vertical hopping (hopV) or (2) continuous lateral hopping (hopL). Participant demographics for each group are given in Table 1.

### 2.2. Instrumentation

Kinematic data were collected with a 10-camera motion capture system (Vicon Motion Systems Ltd., Oxford, UK) sampled at 240 Hz. and kinetic data were obtained from two embedded force platforms, one for each leg (Advanced Medical Technology Inc., Watertown, MA, USA) sampled at 1200 Hz [12]. Calibration of the motion capture cameras was completed via a reflective wand procedure prior to each participant performing the task. The force plates were “zeroed” prior to data collection to set a true 0 N baseline. The accelerometer and HR monitor were factory calibrated. Vertical head acceleration data were obtained via a uniaxial accelerometer (PCB Piezotronics Inc. 52456; Depaw, NY, USA) sampling at 1000 Hz. This accelerometer was fixed to the forehead of each participant’s via a plastic headpiece that was individually fitted to be secured around each participant’s head. A wrist heart rate (HR) monitor (Polar, Polar Electro, Kempele, Finland) sampled at 135 Hz was used for HR analyses.

### 2.3. Procedures

Prior to data collection, this study was approved by the institutional review board at the host university. Following verbal explanation of the study protocol and the opportunity to ask clarifying questions, all participants provided written consent for their participation. Upon signing the written consent form, the research team familiarized the participants with all testing procedures.

Upon familiarization with the study protocol, the HR monitor was strapped to the left wrist of participants. The participants were then instructed to sit quietly for five minutes while their HR was measured. Participants’ maximum heart rate (HR max) was estimated using the following formula: 220 – age. Their target heart rate (HR) for the hopping activity was set to a value of 60% of each participant’s estimated HR max. HR values for each group are included in Table 1. Participants then underwent a standardized warmup, which included “knee hugs”, “quadricep pulls”, “high knees”, “butt kickers”, and body weight squats. After completing the warmup, participants were fitted with reflective markers (B&L Engineering, Santa Ana, CA, USA) used for biomechanical analyses. A set of marker clusters containing four non-collinear markers were placed on the participants’ sacrum, bilaterally on the lateral portion of the participant’s thigh and leg, and bilaterally on each participant’s heel. Static individual markers were placed bilaterally on the participants’ iliac crest, anterior superior iliac spine, greater trochanter, femoral condyles, medial and lateral malleoli, and first and fifth metatarsals. After all markers were placed, participants performed a static calibration trial. They next proceeded to perform their pre-intervention land-and-cut task. Five land-and-cut trials were completed in each direction. After ten pre-intervention land-and-cut trials were completed, participants performed either a continuous lateral hopping task or a continuous vertical hopping task depending on which group they were assigned to. Detailed information about the hopping interventions is described in Section 2.5. Upon completing the hopping intervention, participants’ HR was observed by the primary investigator and each participant noted their level of exertion on a Borg Rate of Perceived Exertion scale from 6 to 20. To ensure that participants were not fatigued from the hopping intervention, their HR must have been less than their previously calculated target HR and they must have been at a rate of perceived exertion level of 9 or below which is categorized as “Very Light” exertion. If the participant had a HR above their target heart rate or they noted a rate of perceived exertion level above 9, then they were instructed to sit and rest. In these instances, the primary investigator reassessed their HR and/or rate of perceived exertion level every thirty seconds until fatigue was ruled out. Fatigue was ruled out when the participant’s HR was below the target HR and they self-reported a rate of perceived exertion at or below level 9. In the instances where the participants were fatigued after the hopping intervention, on average, fatigue was ruled out within one minute after rest was given. After fatigue was ruled out or accommodated for, participants performed another set of land-and-cut trials. A total of ten land-and-cut trials (five in each direction) were completed post-intervention. 

### 2.4. Land-and-Cut Task

Participants began by standing on a 60 cm platform facing a visual stimulus placed approximately 3 m in from of them at eye level when standing on the platform. The platform was 60 cm from the horizontal edge of the force platform. The visual stimulus displayed either a red or blue light that denoted either a cut to the left or a cut to the right, respectively. Upon the visual cue, participants were instructed to step off the box, land on both feet (one foot on each force platform), then perform a 45-degree cutting maneuver to either the left or right as quickly as possible. The limb that performed the cutting maneuver (the stance limb) was the limb of interest for analyzing biomechanical variables. For cutting trials to the left, the right limb was the limb of interest. For cutting trials to the right, the left limb was the limb of interest. The stance limb was chosen as the limb of interest since it was the primary limb used for propulsion and force production during the land-and-cut task. Each participant was allowed as many practice trials as they needed to become familiarized with the land-and-cut task.

### 2.5. Hopping Interventions

Participants in the hopV group were instructed to jump straight up, as high as possible, continuously in place for thirty seconds. They were instructed to jump on every beat of a metronome that was set to a standardized cadence of thirty beats per minute (total of fifteen hopping cycles for every trial of the intervention). Participants in the hopL group were instructed to jump laterally over a tape on the ground continuously for thirty seconds. The tape served as a visual target that participants had to jump over during the lateral hopping task. Participants began by standing directly to the side of the tape and were told that they needed to hop laterally over the tape every time. Similar to the hopV group, they were instructed to jump on every beat of a metronome that was set to a standardized cadence of thirty beats per minute.

### 2.6. Data Analysis

For the land-and-cut maneuvers to both sides, biomechanical variables of interest included peak kinetic and kinematic variables that have been prospectively associated with lower-extremity injury during high-impact loading tasks [5,12,15,17,18,21]. The variables of interest were vertical ground reaction force (force per unit of body weight [BWs]), knee flexion angle (degrees), knee abduction angle (degrees), ankle dorsiflexion angle (degrees), vertical impulse (Newtons × seconds per unit of body weight [(N × s)/BW]), and hip flexion angle (degrees). Knee abduction angle was expressed as a positive value while knee adduction was expressed as a negative value. The remainder of the dependent variables were expressed as positive values. Each kinematic and kinetic dependent variable was assessed during the first 100 ms of ground contact with the force platform, which is consistent with the time frame during which many landing-related injuries occur [22]. Ground contact was defined as the point where the vertical ground reaction force value exceeded 20N on the force platforms. For cutting trials to the left, biomechanical computations were performed on the right limb. For cutting trials to the right, biomechanical computations were performed on the left limb. Kinematic and kinetic biomechanical computations were performed using the Visual 3D software (C-Motion, Germantown, MD, USA), in which marker trajectory and force platform data were smoothed with a fourth-order, low-pass Butterworth filter at 10 Hz and 50 Hz, respectively. Kinetic data were computed using inverse dynamics and normalized to each participant’s weight. During the hopping interventions, head acceleration data were collected using Bioware software (Kistler 4.0; Amherst, NY, USA) at a frequency of 1000 Hz and exported to Microsoft Excel (Redmond, WA, USA) for further analyses. For the analysis of head acceleration magnitude, positive vertical acceleration from each trial of the hopping interventions were collected. Built-in Microsoft excel functions were used to determine peak positive head acceleration values from each hop during the hopping intervention. Peak positive acceleration was selected for analysis as that value typically represented the head acceleration present at impact with the ground after hopping. After the peak positive head acceleration was obtained for all hops in the intervention, the average of those fifteen hops was used for analysis. Lower-extremity kinetics and kinematics were not collected during the hopping interventions.

### 2.7. Statistical Analysis

An independent t-test was performed to compare the head acceleration magnitude between the hopV and hopL groups. A paired t-test was performed for each dependent kinetic and kinematic variable of interest for each limb to compare differences pre- and post-hopping intervention. The alpha level was set to 0.05; however, after using a Bonferroni correction to adjust for multiple tests, the alpha level to achieve significant differences was adjusted to 0.003. For reporting effect size, the absolute value of Cohen’s d was used. Extreme high outlier values for each variable were identified in SPSS Version 27.0 (IBM) as any value greater than the 75th percentile (Quartile 3) + 3 times the interquartile range (IQR) [Quartile 3 value + 3IQR]. Extreme low outlier values for each variable were identified as any value lower than the 25th percentile (Quartile 1) − 3 times the IQR [Quartile 1 value − 3IQR]. If any extreme high and low outlier values were identified, they would be examined by the investigator individually to assess for accuracy in the measurement. Outliers that were deemed to be an error due to inaccurate measurement would be excluded from the analysis. Outliers were assessed by inspection of a boxplot. Normality was assessed using the Shapiro–Wilk normality test for each cell of the design and homogeneity of variances was assessed by Levene’s test. There were no outliers, residuals were normally distributed (*p* > 0.05), and there was homogeneity of variances (*p* > 0.05). 

## 3. Results

The mean, standard deviation, and effect sizes of the within-group comparisons of dependent variables for the hopV group and the hopL group are given in Table 2 and Table 3. None of the *p*-values for the difference in lower-extremity biomechanics pre- and post-intervention within groups met the threshold for statistical significance.

**Figure 1:**
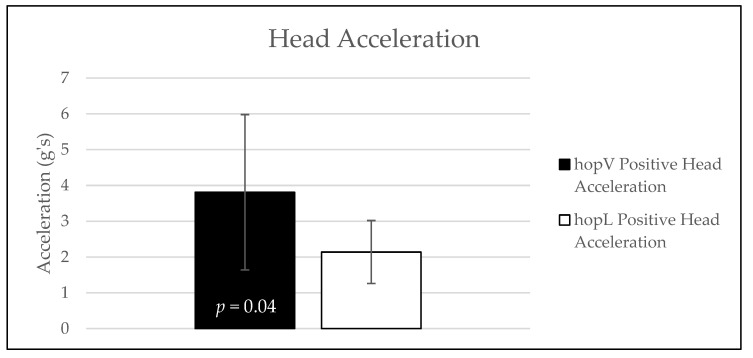
Head acceleration comparisons between groups.

**Figure 2:**
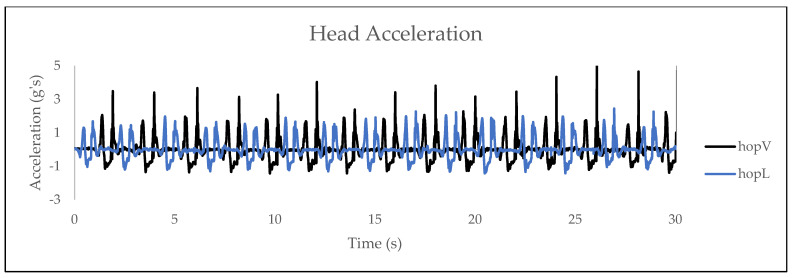
Head acceleration plotted against time for the continuous vertical hopping task and the continuous lateral hopping task.

**Figure 3:**
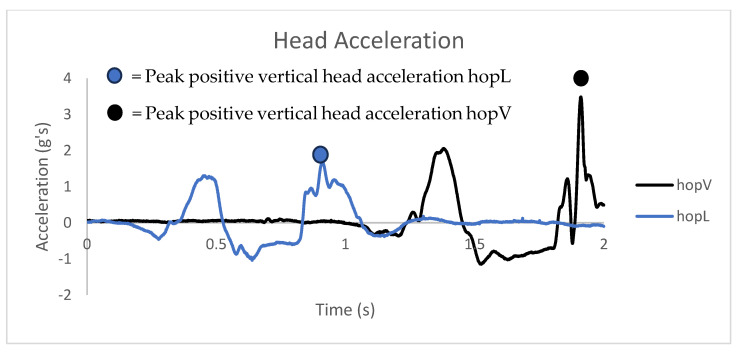
Head acceleration plotted against time for one single hop for each task.

The average head acceleration values and standard deviation for each group are shown in Figure 1. The hopV group exhibited greater positive vertical head acceleration (M = 3.81, SD = 2.17) than the hopL group (M = 2.14, SD = 0.88), with a statistically significant difference (M = 1.67, 95% Confidence Interval [0.06, 3.28], t(18) = 2.256, *p* = 0.04) observed between groups. A visual exemplar of the head acceleration magnitudes exhibited during each hopping task is shown in Figure 2 and Figure 3. The mean, standard deviation, and effect sizes of the post-intervention comparisons between the hopV and hopL groups are shown in Table 4.

Individuals in the hopV group demonstrated greater knee abduction angles during landing (M = 7.5, SD = 3.9) than the hopL group (M = −0.5, SD = 2.6), a statistically significant difference (M = 7.94, 95% Confidence Interval [4.81, 11.06], t(18) = 5.338, *p* < 0.000). 

## 4. Discussion

The overall purpose of this study was to examine the influence of vertical head acceleration values measured during a uniquely directional continuous hopping activity on lower-extremity biomechanics during a land-and-cut task. Additionally, this study served to address a current gap in the literature as to whether there is a relationship between the magnitude of head acceleration and lower-extremity mechanics during landing-related tasks. Lastly, this study assessed whether the induced head acceleration via a continuous hopping task leads to a substantial enough perturbation to the system to elicit a temporary neuromuscular response during subsequent landing tasks. During the hopping interventions, significantly greater positive vertical head acceleration was present in the hopV task compared to the hopL task. This was expected due to the demands of each task. Participants in the hopV group were instructed to jump as high as they possibly could, causing them to land from a higher height. The accommodation strategy utilized to land from a higher height likely translated to increased positive vertical head acceleration to accomplish the task. The magnitude of positive head acceleration obtained from the hopV group was similar to other studies assessing head acceleration during jumping tasks [19,23,24]. The uniqueness in this study lies in the use of head acceleration as an independent variable to explain differences in lower-extremity biomechanics during landing. The prior literature examining head acceleration during landing has examined head acceleration as a dependent variable. This study demonstrated that increased head acceleration can have an influence on lower-extremity biomechanics during high-impact landing tasks. Participants in the hopV group demonstrated a lesser impulse in the right limb in the post-intervention land-and-cut trials and greater knee flexion in both the left and right limbs in the post-intervention land-and-cut trials. Although the within-group differences had a *p*-value less than 0.05 and had large effect sizes, due to the Bonferroni correction for multiple comparisons, the *p*-value obtained did not meet the threshold for statistical significance in this study. There were no significant within-group differences in lower-extremity mechanics during the land-and-cut trials in the hopL group, indicating minimal to no influence of head acceleration on the landing mechanics in this group. When comparing differences in landing mechanics during the land-and-cut trials between groups post-hopping intervention, participants in the hopV group demonstrated greater knee abduction angles in the left limb, less impulse in the right limb, and a greater knee flexion angle in the right limb compared to the hopL group. Like the within-group differences, the between-group differences in impulse and knee flexion angle had a *p*-value less than 0.05 and had large effect sizes. But, due to the Bonferroni correction for multiple comparisons, the *p*-value obtained did not meet the threshold for statistical significance in this study. The only dependent variable that was significantly different after applying a Bonferroni adjustment was the post-intervention left-limb knee abduction angle between groups. During the land-and-cut trials, participants in the hopL group landed with an average frontal plane knee angle that was near the neutral position while participants in the hopV group landed in a more abducted position at the knee joint. Greater knee abduction angles during high-impact landing tasks have been associated with an increased likelihood of anterior cruciate ligament injuries [22,25]. Video analysis of anterior cruciate ligament injuries showed that they tend to occur when knee abduction angles were between 7 degrees and 15 degrees within the first 40 ms after landing [22]. The average knee abduction angles observed in the hopV group post-intervention were within that range, thus raising potential concerns as to the possibility of elevated injury risk. Further research, however, is needed to truly confirm the injury risk implications due to induced head acceleration.

It is important to note that the population in this study were healthy individuals without a history of SRC. Head acceleration during the hopping trials was used as a simulation of the measure of head impact. While the head acceleration values observed during the hopping trials were not nearly substantial enough to achieve the typical threshold to induce an SRC nor adequately simulate a sub-concussive load [19,20], the induced head acceleration still had an influence on the landing strategy adopted by the participants post-intervention. This indicated that the induced head acceleration via the continuous vertical hopping task represented a perturbation to the system that was enough to elicit a temporarily altered neuromuscular response during landing. This suggests that there is utility in inducing head acceleration as a perturbation to the system as a simulation of head impact. Furthermore, if the induced head acceleration from the hopping interventions contributed to altered landing strategies in healthy individuals, causing them to exhibit lower-extremity mechanics conducive to elevated injury risk, it is certainly possible that these effects can be magnified in athletes with a neuromuscular control deficit stemming from SRC. To further validate the use of a hopping intervention to simulate head impact, future studies should directly compare healthy individuals’ landing mechanics after the hopping intervention with the landing mechanics of athletes with a history of SRC. 

### Limitations

While the significant difference in knee abduction angle between the groups may indicate the possibility of increased musculoskeletal injury risk, the non-significant findings in the other dependent variables of interest suggest that lower-extremity landing mechanics and neuromuscular control were mostly similar between the groups. The similarity in landing mechanics and neuromuscular control between and within groups can potentially be attributed to the homogeneity in our population of healthy young adults. Future studies should expand the population of interest to include individuals more at risk for musculoskeletal injury such as youth or adult athletes with an SRC history. Expanding the population to athletes can lead to more generalizable results and may provide further value of the use of the cutting task as athletes may be more familiar with the movement. Furthermore, while this study showed that a continuous vertical hopping task led to a head acceleration magnitude that induced lower-extremity differences during landing, the magnitude of the head acceleration observed was not substantial enough to be deemed a true sub-concussive or concussive load. Additionally, regarding head acceleration, this study only assessed linear acceleration of the head. Most SRCs are caused by angular or rotational accelerations of the head area [19,23], and thus, future studies should seek to compare both linear and angular accelerations of the head.

## 5. Conclusions

Investigating the influence of head acceleration on lower-extremity landing mechanics can have significant implications for injury risk. Furthermore, greater induced head acceleration can contribute to neuromuscular control during high-impact landing tasks. While further research is required to prove the utility in examining head acceleration and its influence on landing mechanics, the results of this study provide some merit regarding using a hopping intervention as a substitute for head impact. Using a hopping task to simulate head impact is safe, efficient, and simple, and can be carried out with minimal equipment. The results of this study provide investigators and clinicians with justification in assessing how induced head acceleration can influence lower-extremity biomechanics during high-impact landing tasks to best reduce the likelihood of individuals sustaining musculoskeletal injuries.

## Figures and Tables

**Table 1:** Participant demographics.

Variable	hopV	hopL
**n**	10 (7F/3M)	10 (7F/3M)
**Age (years)**	23.40 ± 3.78	21.80 ± 2.86
**Height (m)**	1.67 ± 0.10	1.70 ± 0.11
**Mass (kg)**	85.40 ± 47.09	70.30 ± 15.76
**Resting HR (bpm)**	81.33 ± 6.95	80.20 ± 11.80
Target HR (bpm) *	118.77 ± 1.91	118.66 ± 1.90

* Target HR was used as an indicator of fatigue level for each participant during the hopping interventions.

**Table 2:** Within-group comparisons hopV group.

Dependent Variable	Left Limb Pre-Test	Left Limb Post-Test	*p*	Cohen’s d	Right Limb Pre-Test	Right Limb Post-Test	*p*	Cohen’s d
**vGRF (BWs)**	3.02 ± 0.58	3.02 ± 1.12	1.00	0	3.25 ± 0.88	3.17 ± 1.25	0.84	0.07
**Impulse (N × s/BW)**	0.55 ± 0.11	0.71 ± 0.30	0.19	0.45	0.60 ± 0.09	0.54 ± 0.11	0.02 *	0.86
**Dorsiflexion (degrees)**	21.45 ± 9.59	24.34 ± 6.80	0.49	0.23	23.98 ± 5.11	24.96 ± 6.25	0.18	0.46
**Knee Abduction (degrees)**	5.16 ± 3.87	7.48 ± 3.89	0.34	0.32	6.58 ± 5.46	5.71 ± 3.76	0.67	0.14
**Knee Flexion (degrees)**	72.42 ± 11.08	84.93 ± 5.99	0.01 *	1.03	72.40 ± 9.48	82.91 ± 8.31	0.008 *	1.07
**Hip Flexion (degrees)**	78.83 ± 16.03	82.45 ± 17.14	0.67	0.14	79.66 ± 19.48	77.47 ± 16.00	0.27	0.37

* significant at 0.05 level.

**Table 3:** Within-group comparisons hopL group.

Dependent Variable	Left Limb Pre-Test	Left Limb Post-Test	*p*	Cohen’s d	Right Limb Pre-Test	Right Limb Post-Test	*p*	Cohen’s d
**vGRF (BWs)**	3.00 ± 0.64	3.20 ± 1.10	0.67	0.14	3.11 ± 0.84	2.87 ± 0.66	0.14	0.51
**Impulse (N × s/BW)**	0.62 ± 0.09	0.57 ± 0.18	0.52	0.21	0.62 ± 0.06	0.64 ± 0.08	0.59	0.18
**Dorsiflexion (degrees)**	20.41 ± 5.43	21.54 ± 9.97	0.78	0.09	29.19 ± 22.73	22.72 ± 5.89	0.40	0.28
**Knee Abduction (degrees)**	0.95 ± 4.21	−0.46 ± 2.65	0.36	0.31	7.50 ± 5.74	7.70 ± 6.99	0.73	0.11
**Knee Flexion (degrees)**	82.70 ± 14.08	85.65 ± 5.27	0.53	0.21	72.46 ± 9.75	73.66 ± 10.67	0.68	0.14
**Hip Flexion (degrees)**	77.38 ± 11.34	76.13 ± 12.54	0.86	0.06	75.53 ± 10.22	76.54 ± 15.72	0.73	0.11

**Table 4:** Post-intervention comparisons between groups.

Dependent Variable	Left Limb hopV	Left Limb hopL	*p*	Cohen’s d	Right Limb hopV	Right Limb hopL	*p*	Cohen’s d
**vGRF (BWs)**	3.01 ± 1.14	3.21 ± 1.11	0.70	0.17	3.16 ± 1.25	2.88 ± 0.65	0.54	0.28
**Impulse (Ns/BW)**	0.71 ± 0.30	0.57 ± 0.18	0.21	0.58	0.54 ± 0.11	0.65 ± 0.08	0.02 *	1.11
**Dorsiflexion (degrees)**	24.34 ± 6.78	21.54 ± 9.95	0.47	0.33	24.95 ± 6.26	22.73 ± 5.88	0.42	0.37
**Knee Abduction (degrees)**	7.48 ± 3.89	−0.45 ± 2.65	0.000 **	2.39	5.72 ± 3.77	7.71 ± 6.98	0.44	0.36
**Knee Flexion (degrees)**	84.93 ± 5.99	85.65 ± 5.27	0.78	0.13	82.92 ± 8.32	73.66 ± 10.65	0.04 *	0.99
**Hip Flexion (degrees)**	82.43 ± 17.15	76.14 ± 12.53	0.36	0.42	77.46 ± 15.99	76.56 ± 15.71	0.90	0.06

* significant at 0.05 level. ** significant after Bonferroni correction.

## Data Availability

The raw data supporting the conclusions of this article will be made available by the authors on request.
